# Influence of Light Quality During Spreading on the Chemical and Aromatic Profile of Summer Green Tea

**DOI:** 10.3390/ijms27156799

**Published:** 2026-07-29

**Authors:** Xiaojiao Xia, Ling Lin, Fan Xu, Yuzhu Liu, Yueling Zhao, Wei Xu

**Affiliations:** Tea Resources Utilization and Quality Testing Key Laboratory of Sichuan Province, College of Horticulture, Sichuan Agricultural University, Chengdu 611130, China; xxj000220@163.com (X.X.); linling941106@sina.com (L.L.); dylan_37@163.com (F.X.); 13038264811@163.com (Y.L.); zhaoyl@sicau.edu.cn (Y.Z.)

**Keywords:** light quality, spreading, summer green tea, flavor

## Abstract

The use of different light qualities during the spreading stage can influence the flavor of green tea. In this study, freshly harvested leaves were exposed to red (GRL), yellow (GYL), blue (GBL), or natural light (GCK, as the control) throughout spreading. The resulting tea quality was subsequently evaluated through sensory assessment, combined with HS-SPME-GC-MS and UPLC-MS/MS analyses. The results showed that blue light significantly increased free amino acid content and promoted the accumulation of compounds associated with freshness. Yellow light treatment enhanced aroma substances, with 2(5H)-furanone, 5-ethyl-3-hydroxy-4-methyl, benzenemethanethiol, and 3-buten-2-one, 4-(2,6,6-trimethyl-1-cyclohexen-1-yl) detected at the highest levels in this group, contributing to the highest sensory aroma score. In contrast, the GRL group exhibited the lowest levels of aroma compounds, although its sensory score remained relatively high. The GCK group exhibited more pronounced chestnut-like aroma characteristics but received the lowest sensory score. Overall, blue light promotes a fresh and rich taste, whereas yellow light contributes more substantially to the development of aromatic characteristics. These findings indicate that adjusting light quality during the spreading stage can improve the flavor quality, providing a potential strategy for optimizing green tea processing practices.

## 1. Introduction

Green tea, one of the earliest tea categories in China, is prized for its clear bright liquor, green infused leaves, and fresh taste with a sweet aftertaste. However, high temperatures and intense sunlight during summer favor carbon metabolism while suppressing nitrogen metabolism [1], resulting in summer green tea with a weaker aroma and stronger bitterness and astringency. Compared with spring tea, summer tea contains lower levels of free amino acids and L-theanine, but higher contents of catechins and total polyphenols [2], contributing to its more pronounced bitter and astringent taste. Summer tea also exhibits a relatively weak aroma profile, which has been linked to variations in aroma precursor composition [3]. Consequently, it typically shows a harsher taste, less smooth mouthfeel, stronger astringency, and less noticeable aroma. These defects collectively impair sensory quality and diminish consumer acceptance. The high polyphenol-to-amino acid ratio and limited accumulation of volatile aroma compounds under high-temperature and strong-light conditions are the primary causes of these undesirable characteristics, highlighting the urgent need for targeted processing strategies to mitigate quality deterioration. The low utilization of summer tea not only lowers its market value but also results in resource waste and economic losses [4]. Enhancing the quality and value utilization of summer tea is crucial for the sustainable advancement of the tea industry.

The pursuit of higher-quality summer tea through technological innovation in processing remains a central research focus. Previous studies have reported that exposing fresh tea leaves to artificial light accelerates molecular movement, increasing the internal energy and leaf temperature. The generated heat is subsequently transferred to the surrounding environment via convection and conduction, promoting moisture evaporation from the leaves [5]. Moreover, the reduction in leaf moisture content is positively correlated with light intensity [6]. Meanwhile, light treatment can enhance tea aroma quality by regulating metabolic pathways related to volatile compound biosynthesis, including terpene alcohol and linolenic acid metabolism [7]. These metabolic pathways play a crucial role in the generation of aroma precursors and shape the overall aroma characteristics of tea.

Previous studies have shown that blue–violet light promotes the accumulation of nitrogen-containing aroma compounds in fresh tea leaves [8]. Furthermore, yellow-light withering promotes the accumulation of floral volatile compounds, such as nerolidol. The increased abundance of these compounds is associated with improved aroma quality in oolong tea [9]. Red light withering can activate genes related to linalool biosynthesis, which further promotes linalool accumulation and consequently makes the floral and fresh aroma attributes of black tea more pronounced [10]. During the spreading stage of green tea, low-temperature treatment combined with yellow light produced a pronounced fresh aroma accompanied by floral and fruity notes, achieving the highest sensory evaluation score. Compared with natural spreading, this treatment significantly improved aroma quality by increasing the content of terpene alcohol volatiles [11]. Studies have demonstrated that orange-light withering increases the relative abundance of linalool oxides and cedrene, and the enrichment of these aroma compounds contributes to improved aroma quality in black tea [12]. In white tea processing, LED green light treatment during withering can also enhance aroma characteristics [13]. Together, these findings indicate that different light qualities can regulate aroma formation in various tea types during processing.

Most previous studies have focused on black tea and oolong tea, primarily examining the effects of different light qualities during withering with particular attention to volatile compound regulation and pigment transformation. By comparison, relatively few studies have focused on green tea, and research on quality improvement of summer green tea is even more limited. This type of tea typically shows undesirable sensory attributes, including pronounced bitterness and astringency, mainly associated with high polyphenol levels and low amino acid content. Therefore, this study used summer bud green tea of the ‘Mingshan Baihao 131’ cultivar, with shoots harvested at the one-bud-and-two-leaf stage. During spreading, tea leaves were subjected to four light conditions: red light, yellow light, blue light, and natural light as the control. Following the treatments, all samples were processed into green tea using an identical processing protocol. This study comprehensively assessed sensory characteristics, key biochemical constituents, volatile compounds, and non-volatile metabolites, aiming to clarify how different light-quality treatments regulate the flavor characteristics of summer green tea.

## 2. Results

### 2.1. Sensory Characteristics of Green Tea Under Different Light-Quality Conditions

**Figure 1:**
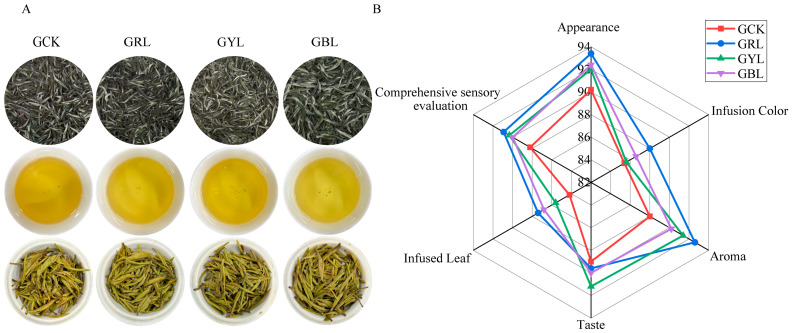
Sensory evaluation of green tea made from summer buds under different light-quality spreading treatments. (**A**) Dry tea appearance, infusion color, and infused leaves of green tea. (**B**) Analysis of appearance, infusion color, aroma, taste, infused leaves, and total scores of the four bud green teas.

The dry tea, infused leaves, and infusion color of the four samples are shown in Figure 1A. Sensory evaluation was performed by scoring appearance, infusion color, aroma, taste, and infused leaves. The overall score was subsequently calculated based on these individual parameters (Figure 1B).

Sensory analysis revealed that light quality primarily influenced the aroma and taste attributes of the green teas. In contrast, dry leaf appearance, infusion color, and infused leaves showed only minor changes. The total sensory scores of all light-exposed groups significantly exceeded that of the GCK (Figure 1B). Among them, GRL scored the highest, with its aroma characterized by bean-like, roasted chestnut-like, and floral notes, although its taste carried a slight astringency. GYL exhibited a smooth, mellow taste with a fresh sensory profile. Its aroma was relatively balanced, with subtle hints of tender chestnut-like and bean-like aromas. The GBL group achieved consistently high and well-balanced rankings across all sensory attributes, with roasted chestnut-like and floral aromas. Previous studies have shown that elevated temperatures accelerate the transformation and volatilization of low-boiling grassy aroma compounds, while promoting the accumulation of high-boiling chestnut-like volatiles [14,15]. This phenomenon may partly explain the frequent occurrence of chestnut-like aroma in green teas subjected to light treatments. Overall, these findings indicate that applying different light-quality conditions during the spreading stage effectively enhances the sensory quality of green tea.

### 2.2. Major Biochemical Components of Green Teas Treated with Different Light Qualities During Spreading

The principal quality-related constituents of summer bud green tea subjected to different light-quality treatments were investigated. The analysis focused on water extract, free amino acids, tea polyphenols, caffeine, and soluble sugar contents (Table 1). Blue-light treatment during spreading significantly elevated both amino acid and soluble sugar contents compared to other light regimes, whereas red light resulted in the lowest amino acid levels. Overall, light quality exerted a pronounced effect on these two chemical constituents. Soluble sugars contribute to sweetness, while amino acid content is a key indicator of green tea freshness and briskness, playing an important role in determining tea taste quality [16,17]. In agreement with the sensory evaluation results, both GBL and GYL treatments enhanced taste quality. In contrast, elevated levels of tea polyphenols, caffeine, and catechins are generally associated with increased bitterness and astringency, whereas insufficient levels of these compounds can leave the tea lacking in flavor [18]. Notably, the contents of water extract, tea polyphenols, and caffeine did not differ significantly among treatments, suggesting that LED light exposure during the spreading stage can improve taste-related attributes without markedly altering the major flavor-related constituents.

Tea contains abundant esterified and non-esterified catechins, which are the major phenolic constituents contributing to taste quality. Among these, epicatechin gallate (ECG) and epigallocatechin gallate (EGCG) are the main esterified catechins, while epigallocatechin (EGC), epicatechin (EC), and catechin (C) belong to the non-esterified group. Esterified catechins are typically associated with strong bitterness and astringency, playing a major role in the astringent perception of tea infusions. In contrast, non-esterified catechins generally exhibit weaker bitterness and astringency and often contribute to a more refreshing aftertaste [19]. As shown in Table 2, the total catechin content across the four samples ranged from 6.85% to 7.59%. Catechin (C) was the predominant non-esterified component in the GCK, GRL, and GYL groups, whereas the GBL group exhibited a markedly lower C content, resulting in the lowest total non-esterified catechins. Esterified catechins accounted for the majority of total catechins in all samples, with EGCG being the most abundant. Notably, the GBL group had an exceptionally high ECG content, which contributed to its highest total esterified catechins. This elevation may be associated with enhanced bitterness and astringency. However, this effect may be counterbalanced by its higher amino acid content (4.80%), leading to a more balanced phenol–amino acid ratio and resulting in a tea infusion described as thick, mellow, fresh, and brisk with a pronounced sweet aftertaste [20].

### 2.3. Influence of Different Light Treatments Applied During the Spreading Stage on Aroma Components of Summer Green Tea

#### 2.3.1. Identification of Aroma Components and Screening of Differential Compounds

**Figure 2:**
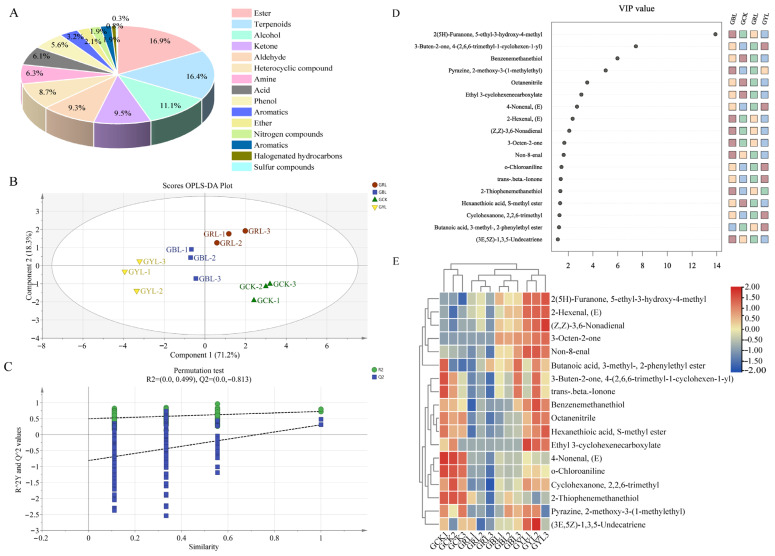
Volatile composition of green tea processed from summer buds under different light-quality spreading conditions. (**A**) Classification and proportion of aroma substances. Note: The sum of percentages is 100.1% due to rounding to one decimal place. (**B**) OPLS-DA of aroma substances. (**C**) OPLS-DA model validation by cross-validation. Green circles represent R^2^Y values, blue squares represent Q^2^ values, and black dashed lines indicate fitted regression lines of the permutation test. (**D**) VIP scores of volatile compounds ranked in descending order. Each black dot represents a compound, and the colored squares indicate the relative abundance across four treatments (GBL, GCK, GRL, GYL). (**E**) Heatmap of cluster analysis of differential metabolites of aroma substances.

A total of 379 volatile constituents were identified in green tea samples subjected to different light-quality treatments using HS-SPME-GC-MS analysis. These belonged to diverse chemical categories, with esters (64), terpenoids (62), and alcohols (43) being the most abundant classes, alongside ketones (36), aldehydes (35), heterocyclic compounds (33), amines (24), acids (23), phenols (21), aromatic hydrocarbons (12), ethers (8), nitrogen-containing compounds (7), hydrocarbons (7), halogenated hydrocarbons (3), and one sulfur-containing compound. The formation of these aroma compounds is known to proceed via multiple processing-related pathways, including Maillard reactions, lipid and carotenoid oxidation, and microbial metabolism [21]. Figure 2A presents the relative proportions of volatile compounds. Aldehydes and esters were the main contributors to green tea aroma, followed by terpenoids at 5.6%. Although sulfur-containing compounds were present at very low levels (0.8%), their extremely low odor thresholds suggest a disproportionately large contribution to overall aroma perception [22].

Orthogonal partial least-squares discriminant analysis (OPLS-DA) was applied to volatile compound datasets to evaluate the influence of different light-quality treatments on aroma profiles. As illustrated in Figure 2B, the first two components of the model explained 71.2% and 18.3% of the total variation in volatile compounds, and clear group separation was observed among the four treatments in the score plot, indicating that different light-quality treatments induced distinguishable differences in volatile profiles. A 200-iteration permutation test was performed to verify model reliability (Figure 2C). After random permutation, the maximum R^2^ was only 0.499, while Q^2^ reached −0.813. All permuted R^2^ and Q^2^ values were markedly lower than those of the original OPLS-DA model, ruling out overfitting and confirming the robustness of our model. Collectively, these multivariate analyses revealed that light-quality conditions during the spreading stage, rather than random variation, drove significant and reproducible changes in green tea volatile composition.

To identify volatile compounds responsible for the separation among different light-quality-treatment groups, VIP analysis was performed based on the OPLS-DA model. As shown in Figure 2D, volatile compounds were ranked in descending order of VIP values. 5-ethyl-3-hydroxy-4-methyl-2(5H)-furanone, a sweet-flavor compound, showed the highest VIP value, followed by 4-(2,6,6-trimethyl-1-cyclohexen-1-yl)-3-buten-2-one, benzenemethanethiol and 2-methoxy-3-(1-methylethyl)pyrazine, all of which are well-documented tea aroma contributors. Notably, 5-ethyl-3-hydroxy-4-methyl-2(5H)-furanone and benzenemethanethiol, both with high VIP values, were enriched in the GYL group, consistent with the ROAV results (Table 2). Additionally, aldehydes including (E)-2-hexenal and (E)-4-nonenal also showed relatively high VIP values and contributed to distinguishing aroma profiles of different treatments. Overall, light-quality conditions during spreading modulated the accumulation of aroma-active compounds, particularly volatiles related to sweet and fresh-scent characteristics.

Volatile constituents with VIP scores exceeding 1 were screened and subjected to hierarchical cluster analysis to further characterize aroma variations among different light-quality treatments. As shown in Figure 2E, vertical clustering revealed that samples from the GRL, GYL, and GBL treatments clustered together, indicating relatively similar aroma profiles among these treatments, which were distinct from the GCK group. Horizontal clustering further highlighted differences in compound distribution among treatments. The GYL group showed higher relative abundances of esters (e.g., butyl butyrate, ethyl 3-methylbutyrate, and methyl anthranilate) and sulfurous compounds (e.g., benzenemethanethiol), along with elevated levels of the sweet aroma compound 5-ethyl-3-hydroxy-4-methyl-2(5H)-furanone. Overall, Yellow-light treatment during spreading enhanced the production of volatile compounds linked to fruity, sweet, and sulfur-containing aroma characteristics. In contrast, the GBL group was enriched in floral-related compounds, including β-ionone, α-ionone, and phenylacetaldehyde, as well as the catechin isomer GCG. Combined with the physicochemical results (Table 1), which showed the highest amino acid content (4.80%) and total esterified catechin content (7.59%) in the GBL group, these findings indicate a coordinated enhancement of freshness- and briskness-related taste attributes and floral aroma characteristics under blue light treatment.

#### 2.3.2. Key Aroma Compounds in Green Tea

The relative odor activity value (rOAV) describes the contribution of each individual volatile compound to the overall aroma profile with higher values indicating stronger sensory relevance. Based on reported criteria [23], compounds with rOAV ≥ 1 were classified as key aroma-active constituents, while those within 0.1 ≤ rOAV < 1 were regarded as important modifiers of aroma characteristics. According to the rOAV results summarized in Table 3, eight volatiles with rOAV > 1 were identified as the main aroma contributors across different light-quality treatments. These included (E)-2-hexenal, 1-nonen-3-one, (Z,Z)-3,6-nonadienal, furan-2-methanol, 2-methoxy-3-(1-methylethyl)pyrazine, including β-ionone. Collectively, these compounds contributed to the fresh and clean aroma profile of bud green tea. Among the key odor-active compounds, the aldehydes (E)-2-hexenal and (Z,Z)-3,6-nonadienal exhibited the highest ROAVs under yellow-light treatment (1.155 and 1.208, respectively). The blue-light group showed slightly lower values (0.962 and 1.061), whereas markedly reduced values were observed in the GRL and GCK treatments. These findings suggest that both GYL and GBL spreading treatments significantly enhanced the production of sweet-smelling volatiles, with the effect being most pronounced under the GYL condition. The sweet aroma compound 5-ethyl-3-hydroxy-4-methyl-2(5H)-furanone also displayed pronounced variation among treatments. The highest rOAV was observed in the GYL group (60.867), followed by GBL (54.524) and GRL (50.548), with all treatment groups exceeding the control (46.194). This indicates that light exposure promoted the formation of sweet aroma compounds, with yellow light showing the strongest effect. Similarly, the sulfur-containing compound benzenemethanethiol exhibited the highest rOAV in the GYL group (7.117), followed by GCK (6.754), GBL (6.360), and GRL (6.046). The floral volatile β-ionone followed a descending order across treatments: GYL (8.561) > GCK (8.269) > GBL (8.159) > GRL (6.982). The GYL and GBL groups were similar to or marginally higher than the control, while the GRL treatment showed a relatively reduced level.

### 2.4. Analysis of Non-Volatile Compounds Under Different Light Qualities

**Figure 3:**
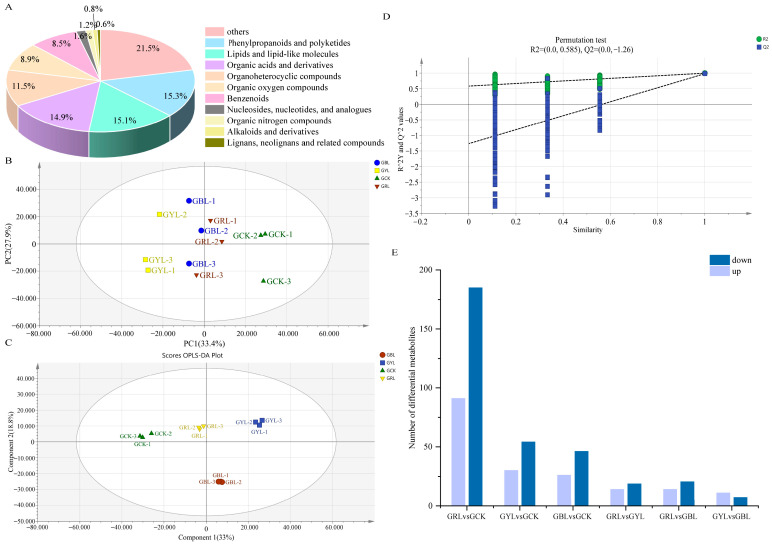
Non-volatile components of summer bud green tea as influenced by light-quality treatments during spreading. (**A**) Classification and proportion of various non-volatile substances. Note: The sum of percentages is 99.9% due to rounding to one decimal place. (**B**) PCA score plot of non-volatile metabolite profiles. (**C**) Score plot of the OPLS-DA model for non-volatile metabolite profiling. (**D**) OPLS-DA model validation by cross-validation. Green circles represent R2Y values, blue squares represent Q2 values, and black dashed lines indicate fitted regression lines of the permutation test. (**E**) Counts of differential non-volatile metabolites.

A total of 2216 non-volatile metabolites were identified in bud green tea samples under four light-quality treatments (GCK, GRL, GYL, and GBL) using UPLC-MS/MS-based untargeted metabolomics combined with multivariate statistical analysis. These metabolites were grouped into 11 categories (Figure 3A). In decreasing order of abundance, they included others (468), phenylpropanoids and polyketides (340), lipids and lipid-like molecules (327), organic acids and derivatives (322), organoheterocyclic compounds (294), organic oxygen compounds (189), benzenoids (184), including nucleotides and nucleotide analogues, organic nitrogen compounds (36), alkaloids and derivatives (26), and lignans, neolignans, and related compounds (13).

PCA revealed clear separation of non-volatile metabolite profiles among samples subjected to different light-quality treatments during spreading (Figure 3B). The first two principal components accounted for 33.4% and 27.9% of the total variance, respectively. The distinct distribution of the four treatment groups suggests pronounced differences in their non-volatile metabolite compositions, with the greatest separation observed between GCK and GYL, suggesting the largest chemical divergence. Notably, GCK samples clustered separately from the GRL, GYL, and GBL groups.

Orthogonal partial least-squares discriminant analysis (OPLS-DA) was performed to filter out variations unrelated to treatments and to screen differential metabolites under different light-quality conditions. As shown in Figure 3C, predictive component 1 and orthogonal component 2 separately accounted for 33% and 18.8% of the total variance of metabolite profiles. The four experimental groups exhibited clear separation in the OPLS-DA score plot, indicating that this model could effectively discriminate samples from different light-quality treatments. Specifically, GBL samples clustered in the lower-left area, GRL samples were distributed in the upper-left quadrant, GYL samples on the right side, and GCK samples were mainly distributed in the left-central area, which was consistent with the PCA results (Figure 3B). A 200-iteration permutation test was subsequently performed to validate model reliability (Figure 3D). The results showed a Q^2^ intercept of −1.26, with all permuted R^2^ and Q^2^ values markedly lower than those of the original model. Additionally, the R^2^ intercept (0.585) exceeded the Q^2^ intercept, collectively confirming that the established OPLS-DA model had high reliability without overfitting.

Differential metabolites were identified using the following criteria: VIP > 1, fold change (FC) > 1.5 or FC < 0.67, and *p*-value < 0.05. The number of up- and downregulated metabolites varied among pairwise comparisons. For GCK vs. GRL, 91 metabolites were upregulated and 208 were downregulated. For GCK vs. GYL, 30 metabolites were upregulated and 61 were downregulated. For GCK vs. GBL, 26 metabolites were upregulated and 52 were downregulated. For GYL vs. GBL, 11 metabolites were upregulated and 8 were downregulated. For GBL vs. GRL, 14 metabolites were upregulated and 23 were downregulated. For GYL vs. GRL, 14 metabolites were upregulated and 21 were downregulated. Metabolites with VIP > 1, FC > 1.5, and *p* < 0.05 were classified as upregulated, whereas those with VIP > 1, FC < 0.67, and *p* < 0.05 were considered downregulated. The overall distribution of differential metabolites across all comparisons is presented in Figure 3E.

**Figure 4:**
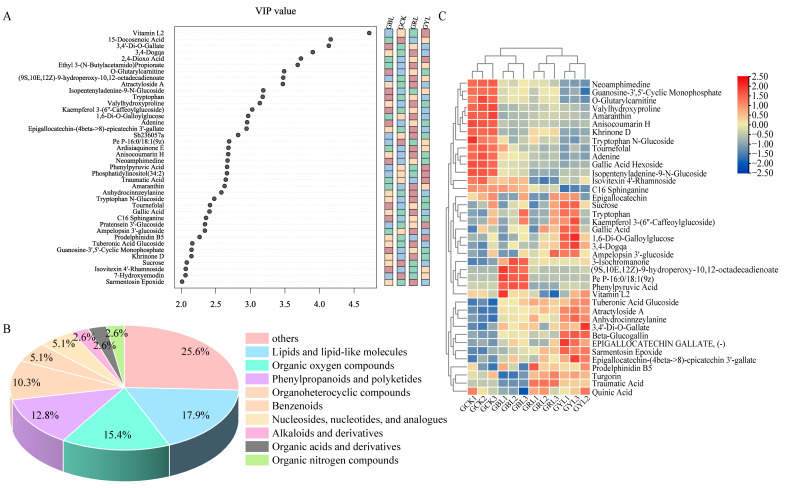
Effects of Light-Quality Treatments during Spreading on Non-Volatile Metabolites in Summer Bud Green Tea. (**A**). VIP (Variable Importance in Projection) scores of the 40 differential metabolites (VIP > 2) in the OPLS-DA model. Metabolites are ranked by VIP values in descending order. Each black dot represents a compound, and the colored squares indicate the relative abundance across four treatments (GBL, GCK, GRL, GYL). (**B**) Chemical class distribution of differential non-volatile metabolites. (**C**) Hierarchical cluster analysis heatmap of differential non-volatile metabolites.

To further identify the key metabolites driving group separation, variable importance in projection (VIP) analysis was performed based on the OPLS-DA model. As shown in Figure 4A, all 40 differential metabolites with VIP > 2 were ranked in descending order. Vitamin L2 exhibited the highest VIP value, followed by 15-docosenoic acid and 3,4′-di-O-gallate, which were the top three contributors to the discrimination of the four experimental groups. Notably, many high-VIP metabolites were associated with phenylpropanoid and flavonoid biosynthesis, including kaempferol 3-(6″-caffeoylglucose), 1,6-di-O-galloylglucose, and epigallocatechin-(4β → 8)-epicatechin 3′-gallate. Additionally, tryptophan-related metabolites also displayed relatively high VIP values, indicating that amino acid metabolism was markedly affected by light-quality conditions during spreading.

To evaluate the effect of light quality during spreading on non-volatile metabolites in green tea, significantly altered metabolites were screened using OPLS-DA with VIP > 2 and *p* < 0.05. A total of 39 metabolites met these criteria and were classified into 10 chemical classes (Figure 4B). In decreasing order of abundance, these included others (10), lipids and lipid-like molecules (7), organic oxygen compounds (6), phenylpropanoids and polyketides (5), organoheterocyclic compounds (4), benzenoids (2), nucleotides and nucleotide analogues (2), along with single representatives from alkaloids and derivatives, organic acids and derivatives, and organic nitrogen compounds.

Figure 4C presents a hierarchical clustering heatmap depicting the differential accumulation of non-volatile metabolites across the GCK, GRL, GYL, and GBL groups. Vertical clustering showed that the light-treated groups (GRL, GYL, and GBL) were clearly separated from the GCK group, indicating that light quality is the primary factor driving the non-volatile metabolome. Furthermore, the GYL and GBL groups clustered together, whereas the GRL group formed a distinct branch, suggesting that the metabolic profiles of GYL and GBL were more similar to each other, while GRL exhibited unique metabolic characteristics. The three biological replicates within each group clustered closely together, indicating good experimental reproducibility and reliable data quality.

Horizontal clustering revealed that the blue-light treatment group displayed extensive red high-expression regions in the heatmap, indicating the enrichment of multiple metabolites, including phenolic acids and hydrolyzable tannins, such as gallic acid and its glycosides, epigallocatechin gallate (EGCG), 1,6-di-O-galloylglucose, and β-glucogallin. Amino acids and their derivatives, including tryptophan and its glycosylated forms, along with phenylpyruvic acid, were also detected. These results suggest that exposure to blue light significantly promotes the accumulation of metabolites associated with the phenylpropanoid biosynthetic pathway. In agreement, the GBL group exhibited the highest amino acid content (4.80%) and the greatest total ester-type catechin content (7.59%) in the biochemical assays, further corroborating the metabolomic observations.

The GYL group also displayed prominent red regions in the heatmap, indicating elevated levels of specific metabolites, primarily traumatic acid and dipeptides such as valyl hydroxyproline. The accumulation of traumatic acid suggests enhanced fatty acid metabolic activity during yellow-light spreading. Unsaturated fatty acids serve as important precursors for aliphatic aroma compounds containing six to ten carbon atoms [24], which is consistent with the aroma analysis results showing that the GYL group had the highest levels of fresh and green aldehydes, including (E)-2-hexenal and (Z,Z)-3,6-nonadienal.

The GRL group displayed a distinct metabolic profile in the heatmap, differing from both the other light-treated groups (GYL, GBL) and the GCK group. Unlike GYL and GBL, GRL did not exhibit extensive high- or low-expression regions but showed selective upregulation of specific metabolites. Notably, adenine was relatively elevated in GRL, whereas intermediates of the tricarboxylic acid cycle, such as aconitic acid, were comparatively low. Aconitic acid is an organic acid contributing to astringency [25]. This metabolic pattern is consistent with an inhibitory effect of red light on primary metabolism, particularly reflected in the widespread downregulation of amino acid and energy metabolism pathways.

The GCK group displayed prominent low-expression regions (blue) for multiple metabolites in the heatmap, in sharp contrast to the light-treated groups. This pattern further supports the notion that light-quality treatments during spreading activate metabolic activity in green tea leaves.

**Figure 5:**
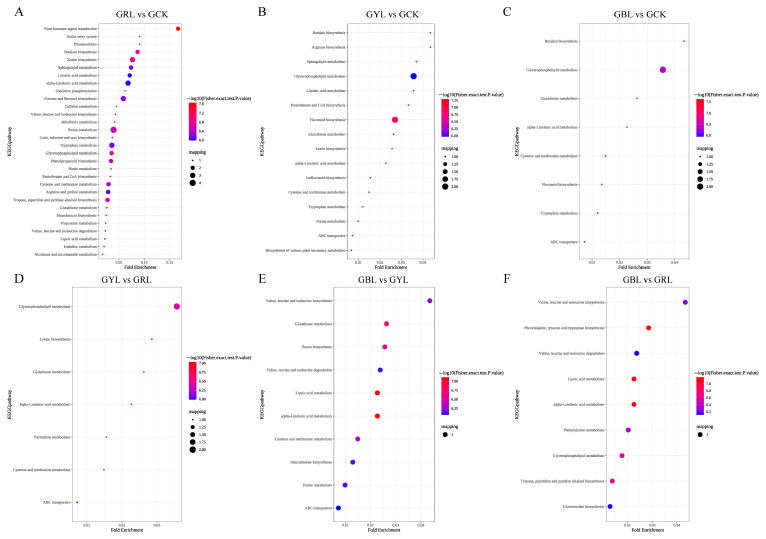
KEGG pathway enrichment analysis of differential non-volatile metabolites across different comparison groups. Bubble size represents the number of metabolites annotated to each pathway, and bubble color indicates the significance level of enrichment. (**A**) GRL vs. GCK; (**B**) GYL vs. GCK; (**C**) GBL vs. GCK; (**D**) GYL vs. GRL; (**E**) GBL vs. GYL; (**F**) GBL vs. GRL.

To further elucidate the biological pathways associated with the differential metabolites, KEGG enrichment analysis was performed on the differential non-volatile metabolites from all comparison groups (Figure 5). As shown in Figure 5, the differential metabolites in the GRL vs. GCK comparison were predominantly enriched in pathways associated with flavonoid biosynthesis, alpha-linolenic acid metabolism, and glutathione metabolism; those in the GYL vs. GCK comparison were mainly enriched in flavonoid biosynthesis, tryptophan metabolism, and cysteine and methionine metabolism; and those in the GBL vs. GCK comparison were mainly enriched in flavonoid biosynthesis, alpha-linolenic acid metabolism, tryptophan metabolism, glutathione metabolism, glycerophospholipid metabolism, as well as cysteine and methionine metabolism. The enrichment results for the other pairwise comparisons (GYL vs. GRL, GBL vs. GYL, and GBL vs. GRL) similarly indicated that flavonoid biosynthesis and amino acid metabolism-related pathways were the core enriched pathways for differential metabolites. Notably, the flavonoid biosynthesis pathway was enriched across all comparison groups, with particularly pronounced enrichment in the GCK-referenced groups, suggesting that different light qualities can modulate the phenolic composition of summer green tea through the regulation of flavonoid metabolism. Among these, the GBL vs. GCK group exhibited the highest −log_10_(*p*-value) for the flavonoid biosynthesis pathway, indicating the strongest enrichment significance. This finding is consistent with the significantly elevated levels of esterified catechins (ECG and GCG) observed in the GBL group (Table 2), further supporting the prominent role of blue light in promoting flavonoid accumulation. The significant enrichment of alpha-linolenic acid metabolism and tryptophan metabolism pathways implies that light quality may also regulate the synthesis of fatty acid derivatives and indole compounds, both of which serve as important precursors for tea aroma formation. Collectively, these results demonstrate that different light qualities synergistically modulate the taste and aroma quality of summer green tea through the coordinated regulation of multiple pathways, including flavonoid metabolism, amino acid metabolism, and fatty acid metabolism, further underscoring that light quality management during the spreading stage represents an effective strategy for improving summer green tea quality.

## 3. Discussion

The present study demonstrated that light quality applied during the spreading-withering stage exerted differential regulatory effects on taste and aroma quality of post-harvest summer green tea leaves. Blue light most effectively promoted the accumulation of esterified catechins and free amino acids, contributing to a balanced flavor profile; yellow light comprehensively improved both the taste and aroma, whereas red light enhanced aroma quality but showed limited effects on taste-related metabolites.

KEGG enrichment analysis revealed that the flavonoid biosynthesis pathway was significantly enriched across all comparison groups, with the most pronounced enrichment in the GBL vs. GCK comparison, which is consistent with the elevated levels of EGCG, ECG, and GCG observed in the GBL group. Previous studies have confirmed that blue light can activate flavonoid metabolism and reshape carbon–nitrogen allocation in tea plants [26,27], and blue-light-dependent epigenetic regulation has also been reported to participate in modulating catechin accumulation [28]. Although these findings were derived from intact growing tea seedlings rather than detached withering leaves, the physiological responses to blue light still partially account for the increased flavonoid contents observed under blue-light withering in our study.

The GBL group exhibited the highest free amino acid content (4.80%), which, together with elevated levels of esterified catechins, contributed to a well-balanced phenol–amino acid ratio. Blue light signals are known to coordinate carbon–nitrogen metabolism in tea plants [28]; notably, even in detached leaves, blue-light-triggered metabolic readjustment facilitated the concomitant accumulation of flavonoids and amino acids, imparting a full mouthfeel and a fresh, mellow, sweet aftertaste.

The elevated levels of fresh and green aldehydes, such as (E)-2-hexenal and (Z,Z)-3,6-nonadienal, in the GYL group are closely associated with fatty acid metabolism. The accumulation of traumatic acid suggested enhanced fatty acid metabolic activity under yellow light, thereby promoting the generation of fatty acid-derived volatiles [29].

Different light qualities induced distinct metabolic shifts. Red light was less conducive to flavonoid biosynthesis but appeared to redirect carbon flux toward terpene-related metabolism [30], explaining the highest aroma score observed in the GRL group despite lower catechin content. Yellow light simultaneously activated both flavonoid and amino acid pathways, and fatty acid-derived volatiles accumulated substantially during yellow-light withering, contributing to a balanced taste and aroma profile.

Collectively, different light-quality treatments triggered carbon–nitrogen redistribution in detached tea leaves during post-harvest withering: blue light promoted flavonoid and amino acid accumulation, red light channeled carbon allocation toward aroma-related metabolites, and yellow light balanced both taste and aroma pathways.

Several limitations of this study should be acknowledged. First, only a single cultivar was used, which may limit the generalizability of the findings. Second, the experiment was conducted under controlled indoor light conditions, which differ from natural environments. ROAV calculations relied on thresholds from online databases, which may not fully account for matrix effects in tea infusions; light intensity was recorded in lux rather than photosynthetic photon flux density (PPFD), as lux does not accurately reflect photon energy absorbed by tea plants. Despite these constraints, this study provides a theoretical basis for light-quality regulation of tea quality during withering.

For practical processing applications, GYL treatment is recommended for aroma-oriented green teas to enhance sweet, floral, and fruity notes; GBL treatment is preferred for fresh-taste-oriented green teas to improve umami and mellow mouthfeel; GRL treatment may be considered for products where aroma quality is prioritized over taste.

## 4. Materials and Methods

### 4.1. Materials and Reagents

The sources of the chemical reagents employed in this study are as follows: sodium carbonate was obtained from Hongdakang Technology (Chengdu, China); concentrated hydrochloric acid and sulfuric acid were purchased from Kelong Chemical Reagent (Chengdu, China); sodium chloride (analytical grade) was supplied by Sinopharm (Beijing, China); acetonitrile and acetic acid (chromatographic grade, purity ≥ 99%) were acquired from Haoboyou Technology (Chengdu, China); anthrone was sourced from Baishibo Biotechnology (Chengdu, China); and ninhydrin, disodium hydrogen phosphate, dipotassium hydrogen phosphate, basic lead acetate, and aluminum chloride were purchased from Jinshan Chemical (Chengdu, China); gallic acid, methanol, ethanol, sodium bicarbonate, ethyl acetate, oxalic acid, and n-butanol were also obtained from Jinshan Chemical (Chengdu, China).

### 4.2. Tea Sample Preparation

On 8 June 2025, fresh tea leaves of the cultivar ‘Mingshan Baihao 131’ (4.0 kg) were obtained from a local tea plantation and immediately processed into green tea in Ya’an City, Sichuan Province, China. The experiment was conducted indoors, with natural light serving as the control (GCK). Three LED light sources with different wavelengths were applied: red light (700 nm), yellow light (595 nm), and blue light (480 nm). During the spreading process, the light intensity at the sample surface was adjusted to 1500 lx using integrated LED T5 tube lights (Model: LS-T5Y-18W-A; Manufacturer: LEESA, Virginia Beach, VA, USA; Power: 28 W; Length: 1.2 m), with the light source positioned 35 cm above the sample surface. Immediately after harvest, the leaves were randomly divided into four batches of 1 kg each. The processing procedure consisted of a 6-h light treatment during spreading, after which the leaves were subjected to fixation, shaping, and drying. Fixation was performed manually using a pan-fixation machine (Shanfeng Tea Machinery Co., Ltd., Mingshan District, Ya’an City, Sichuan, China) at 250 ± 10 °C for 10 min. Shaping was performed using a 6CL-6580 machine (Shanfeng Tea Machinery Co., Ltd., Mingshan District, Ya’an City, Sichuan, China) at 100 °C for 10 min, followed by cooling at room temperature for an additional 10 min. Aroma enhancement and drying were carried out using a DL16 tea baking and aroma-enhancing machine (Shanfeng Tea Machinery Co., Ltd., Mingshan District, Ya’an City, China). Initial drying was conducted at 110 °C with a leaf layer thickness of 1 cm for 10 min, at which point the leaves exhibited a slightly coarse surface texture. Samples were then cooled and allowed to re-moisten for 30 min until soft. Final drying was performed at 70 °C with a leaf layer thickness of 1 cm until complete dryness was achieved, defined as the leaves being easily crumbled into powder by hand. After processing, all samples were stored at 4 °C prior to further analysis.

### 4.3. Sensory Evaluation

Sensory evaluation was conducted by five certified tea tasters (National Vocational Qualification Level 3, China) in accordance with the Chinese National Standard GB/T 23776-2018 [31], Methodology for Sensory Evaluation of Tea. For each sample, 3.0 g of tea leaves was infused with 150 mL of boiling water for 4 min. The infusion was then separated from the tea leaves, whereas the infused leaves were retained in the cup for evaluation. The tasters evaluated the samples in a fixed sequence: infusion color, aroma, taste, and infused leaf appearance. Sensory quality was scored using a 100-point scale. All evaluations were conducted independently under blinded conditions, and the final sensory score for each sample was calculated as the average of the five individual scores.

### 4.4. Analysis of Major Physicochemical Components

Moisture content and water-soluble extracts were determined according to GB/T 8305-2013 [32]. Total free amino acids were measured using a previously described method with slight modifications [33]. Total polyphenol content was analyzed via the Folin–Ciocalteu method [34], whereas soluble sugars were quantified using the anthrone–sulfuric acid assay [35]. Catechins and caffeine were simultaneously quantified using an Agilent HPLC system (Agilent Technologies, Santa Clara, CA, USA). Chromatographic separation was performed on an Athena C18-WP column (Shanghai Anpel Experimental Technology Co., Ltd., Shanghai, China). The mobile phase consisted of 0.2% acetic acid aqueous solution (A), acetonitrile (B), and methanol (C). The injection volume was 1 mL, and detection was carried out at 278 nm.

### 4.5. HS-SPME-GC-MS Analysis

#### 4.5.1. Extraction of Volatile Compounds

Headspace solid-phase microextraction (HS-SPME) was employed to isolate volatile compounds from tea samples. For each analysis, approximately 200 mg of sample was accurately weighed into a headspace vial. Saturated NaCl and an internal standard were then added, and the mixture was equilibrated at 60 °C for 5 min. Volatile compounds were adsorbed onto a 120-μm DVB/CWR/PDMS fiber for 15 min. The fiber was subsequently introduced into the GC injection port and thermally desorbed at 250 °C for 5 min. The released volatiles were then separated and identified by GC-MS.

#### 4.5.2. Qualitative and Quantitative Methods

A pooled QC sample was injected at regular intervals throughout each analytical run to monitor instrument stability and response reproducibility. QC data were excluded from subsequent differential analysis and result interpretation.

GC-MS analysis was performed on a DB-5MS column (30 m × 0.25 mm × 0.25 μm, Agilent J&W Scientific, Folsom, CA, USA) with helium (purity ≥ 99.999%) as the carrier gas at a flow rate of 1.2 mL/min. Samples were injected in splitless mode at 250 °C. The oven temperature was programmed from 40 °C to 280 °C. An electron impact (EI) source was employed for MS detection, with the ion source and quadrupole temperatures set at 230 °C and 150 °C, respectively. The interface temperature was maintained at 280 °C. The electron energy was fixed at 70 eV. Data acquisition was performed in selected ion monitoring (SIM) mode for both qualitative and quantitative analyses.

After background signals were removed, compounds were identified by comparing their retention times and characteristic ions with those of corresponding reference standards according to their elution order [36]. Peak integration and quantitative calibration were performed using selected quantifier ions to ensure analytical accuracy. Following data acquisition, raw mass spectra were processed with MassHunter software (version 10.0, Agilent Technologies, Santa Clara, CA, USA) for peak integration and calibration. Qualitative and quantitative metabolite annotation was carried out against the custom-built library of Sanshu Biotechnology (Sanshu Biotechnology Co., Ltd., Shanghai, China). In addition, the analysis was conducted using the web-based tool. Odor descriptions and odor thresholds of the volatile compounds were obtained from publicly accessible databases, including The Good Scents Company (http://www.thegoodscentscompany.com/ accessed on 15–31 July 2025), Perflavory (http://perflavory.com/, accessed on 15–31 July 2025), Odour Database (http://www.odour.org.uk/, accessed on 15–31 July 2025), and Food Flavor Laboratory (http://foodflavorlab.cn/, accessed on 15–31 July 2025).

#### 4.5.3. ROAV Analysis

The odor activity value (OAV) was used to evaluate the contribution of volatile compounds to the aroma profile of green tea under different light-quality treatments. Also referred to as flavor units (FU), the OAV is defined as the ratio of the absolute concentration of a volatile compound (C) to its corresponding odor threshold (T).

(1) $${\mathrm{OAV}}_{i}=\frac{C_{i}}{T_{i}}$$

Given the large diversity of volatile compounds present in tea samples, complete absolute quantification is both time-consuming and costly. To address this limitation, relative odor activity values (ROAVs) were employed to estimate the relative contribution of individual volatile compounds to the overall aroma profile.

The relative concentration was expressed as:

*C_i_*% = *f*·*C_i_* (2)

The compound exhibiting the maximum OAV was selected as the reference standard and assigned a value of 100. The ROAVs of the remaining compounds were then calculated relative to this reference compound.

(3) $${\mathrm{ROAV}}_{i}=100\;\times \;\frac{{OAV}_{i}}{{OAV}_{max}}$$

where OAV_max_ represents the highest OAV among all identified compounds. All identified compounds showed ROAVs ≤ 100, with higher values indicating a greater contribution to the overall aroma profile. In this study, compounds with ROAV ≥ 1 were classified as key aroma-active constituents, whereas those with 0.1 ≤ ROAV < 1 were considered important contributors to aroma development [37].

### 4.6. Untargeted Non-Volatile Compound Analysis

#### 4.6.1. Sample Extraction

For metabolite extraction, samples were thawed at 4 °C prior to processing. The procedure involved sample weighing, solvent extraction, vortex-mixing, low-temperature ultrasonication for 30 min, and centrifugation at 12,000 rpm and 4 °C for 10 min. Protein precipitation was then induced by storing the supernatant at −20 °C for 1 h, followed by a second centrifugation step under the same conditions. The resulting supernatant was subsequently evaporated to dryness under vacuum. The dried residue was redissolved in 200 μL of 30% acetonitrile, vortexed, and centrifuged at 14,000 rpm for 15 min at 4 °C. Finally, the supernatant was transferred into an autosampler vial for instrumental analysis.

#### 4.6.2. Analytical Conditions

LC separation was carried out using the following gradient program: 0–1.0 min, 100% A/0% B; 1.0–4.0 min, linear gradient to 40% A/60% B; 4.0–6.5 min, linear gradient to 5% A/95% B; 6.5–6.6 min, return to 100% A/0% B; followed by re-equilibration with 100% A/0% B until 8.0 min.

### 4.7. Data Statistics and Analysis

All experiments were conducted with three biological replicates, and the mean values were used for subsequent data analysis. One-way analysis of variance (ANOVA) was performed to assess statistical differences using IBM SPSS Statistics (version 27). Volatile and non-volatile metabolite profiles were visualized using Origin 2024. Multivariate statistical analyses, including principal component analysis (PCA) and orthogonal partial least squares discriminant analysis (OPLS-DA), were carried out with SIMCA 14.1.

## Figures and Tables

**Table 1:** Biochemical profiles of summer green tea exposed to different spreading conditions in terms of light quality.

Content (%)	GCK	GRL	GYL	GBL
Moisture content	4.30 ± 0.08 b	3.97 ± 0.31 b	3.48 ± 0.17 c	4.87 ± 0.20 a
water extract	46.08 ± 0.35 b	46.66 ± 0.54 b	46.68 ± 0.58 b	48.55 ± 0.69 a
amino acid	4.14 ± 0.39 bc	3.83 ± 0.21 c	4.41 ± 0.20 ab	4.80 ± 0.12 a
tea polyphenols	20.05 ± 0.42 a	18.99 ± 0.37 a	19.13 ± 0.39 a	19.18 ± 0.90 a
soluble sugar	4.29 ± 0.01 a	4.27 ± 0.01 c	4.28 ± 0.01 b	4.30 ± 0.01 a
caffeine	4.04 ± 1.12 a	3.91 ± 0.24 a	4.16 ± 0.26 a	3.92 ± 0.09 a

Note: different letters within the same row indicate significant differences (*p* < 0.05).

**Table 2:** Catechin profiles of summer green tea exposed to different light-quality treatments during spreading.

Content (%)	GCK	GRL	GYL	GBL
EGC	0.12 ± 0.01 a	0.12 ± 0.01 a	0.12 ± 0.01 a	0.11 ± 0.01 a
C	1.25 ± 0.06 ab	1.40 ± 0.37 a	1.40 ± 0.44 a	0.76 ± 0.08 b
EC	0.12 ± 0.02 a	0.12 ± 0.01 a	0.11 ± 0.01 a	0.10 ± 0.01 a
EGCG	4.81 ± 0.33 a	4.57 ± 0.31 a	4.69 ± 0.22 a	4.29 ± 0.13 a
GCG	0.13 ± 0.01 c	0.37 ± 0.01 b	0.40 ± 0.01 a	0.38 ± 0.01 b
CG	0.11 ± 0.01 a	0.09 ± 0.01 b	0.09 ± 0.01 b	0.09 ± 0.01 b
ECG	0.31 ± 0.01 b	0.31 ± 0.01 b	0.32 ± 0.01 b	1.86 ± 0.08 a

Note: different letters within the same row indicate significant differences (*p* < 0.05).

**Table 3:** ROAV of volatile compounds under different light-quality treatments.

Volatile Components	Odor (Main)	RI	Threshold (μg/kg)	ROAV			
				GCK	GRL	GYL	GBL
2-Hexenal, (E)	green	853.2	3.100	0.740	0.838	1.155	0.962
1-Nonen-3-one	mushroom	1076	0.001	100	100	100	100
(Z,Z)-3,6-Nonadienal	green	1100	0.050	0.888	0.927	1.208	1.061
Pyrazine, 2-methoxy-3-(1-methylethyl)	nutty	1097	0.002	6.790	6.007	6.581	6.584
2(5H)-Furanone, 5-ethyl-3-hydroxy-4-methyl	sweet	1195	0.002	46.194	50.548	60.867	54.524
Benzenemethanethiol	minty	1092	0.004	6.754	6.046	7.117	6.360
Octanenitrile	green	1081	0.130	2.465	2.214	2.472	2.266
3-Buten-2-one,4-(2,6,6-trimethyl-1-cyclohexen-1-yl)	floral	1491	0.007	8.269	6.982	8.561	8.159

Note: The compound with the highest OAV in each treatment was selected as the reference standard, and its ROAV was normalized to 100. 1-Nonen-3-one exhibited the highest OAV across all four treatments (GCK, GYL, GRL, and GBL); therefore, its ROAV value was consistently 100 in all groups. Volatile constituents with ROAV ≥ 1 were regarded as the primary contributors to the aroma profile of the analyzed samples.

## Data Availability

The datasets used and/or analysed during the current study are available from the corresponding author on reasonable request.

## Abbreviations

The following abbreviations are used in this manuscript:

ROAV	Relative Odor Activity Value
ECG	Epicatechin Gallate
EGCG	Epigallocatechin Gallate
EC	Epicatechin
C	Catechin
PCA	Principal Component Analysis
OPLS-DA	Orthogonal Partial Least Squares Discriminant Analysis
VIP	Variable Importance in Projection
PPFD	Photosynthetic photon flux density
